# Preparation of 3-Iodo-2-propargyl-butyl-carbamate-Loaded Microcapsules for Long-Term Mold Resistance in Bamboo

**DOI:** 10.3390/polym17050679

**Published:** 2025-03-04

**Authors:** Gege Bao, Lu He, Xiaofeng Zhang, Xi Yu, Jingpeng Li, Daochun Qin

**Affiliations:** 1 International Centre for Bamboo and Rattan, Beijing 100102, China; baophd1@outlook.com (G.B.); 18381378592@163.com (L.H.); zxf15717483927@163.com (X.Z.); yuxi_677@foxmail.com (X.Y.); 2 Key Laboratory of National Forestry and Grassland Administration for Bamboo and Rattan Science & Technology, Beijing 100102, China; 3 Key Laboratory of High Efficient Processing of Bamboo of Zhejiang Province, China National Bamboo Research Center, Hangzhou 310012, China; 4 Sanya Research Base, International Centre for Bamboo and Rattan, Sanya 572000, China

**Keywords:** bamboo, microcapsules, melamine-formaldehyde resin, iodo-2-propargyl-butyl-carbamate, anti-mold

## Abstract

Bamboo, recognized as a nutrient-dense biomass material, exhibits a high susceptibility to mold infestations, which can result in discoloration and a notable decrease in longevity, thereby posing potential health risks to humans. In this study, melamine-formaldehyde resin (MFR) was utilized to load 3-iodo-2-propargyl-butyl-carbamate (IPBC) via in situ polymerization, resulting in the preparation of microcapsules suitable for anti-mold protection of bamboo. The mold resistance of *Aspergillus niger*, *Trichoderma viride*, and *Penicillium citrinum* were evaluated. A scanning electron microscope (SEM), transmission electron microscope (TEM), Fourier-transform infrared spectrometer (FTIR), and thermogravimetric analysis (TG) were used to characterize and analyze the formation process, surface morphology, structural composition, and thermal stability of the microcapsules. The effects of different surfactants (Span 80, Tween 80, SDBS, SDS, GA) on the microscopic morphology of the anti-mold microcapsules were investigated. The results show that microcapsules prepared with Tween 80 as the surfactant exhibited good mold resistance. After coating MFR with IPBC, the drug loading of I-MFR is 20%, with an encapsulation efficiency of 80%, demonstrating excellent anti-mold performance. The microcapsules show favorable anti-mold performance and have broad application prospects in bamboo protection.

## 1. Introduction

Bamboo, recognized as “green gold”, represents a critical ecological, industrial, and biomass resource that plays a pivotal role in the global carbon cycle [1,2,3,4]. China, possessing the world’s most abundant bamboo resources, has developed a bamboo industry with an annual output value of approximately CNY 320 billion, emerging as a promising forestry sector [5,6]. Bamboo is characterized by rapid growth, early maturity, high yield, and sustainable utilization, allowing for harvesting within 3–4 years, making it an effective alternative to alleviating timber resource scarcity [7,8]. However, bamboo products are susceptible to mold attack due to high starch content, leading to deformation and degradation that significantly compromise their durability and esthetic value [9,10]. With increasing environmental awareness, traditional bamboo preservatives such as creosote and pentachlorophenol have been progressively phased out due to their adverse environmental and human health impacts [11]. Consequently, eco-friendly antifungal agents and treatment processes tailored to bamboo characteristics are rapidly developing.

Notably, 3-iodo-2-propargyl-butyl-carbamate (IPBC), a novel broad-spectrum fungicide, has been extensively utilized in industrial mold prevention, cosmetic preservation, and bamboo protection [12,13,14]. Although IPBC exhibits significant antifungal effects, several issues commonly arise during its practical use: (1) limited thermal stability; (2) it is easily UV-decomposable; and (3) leaching, which all pose the risk of environmental pollution and human sensitization [15,16,17,18,19]. These limitations significantly hinder IPBC use in protective applications. Maintaining IPBC anti-mold efficacy while enhancing thermal stability, UV resistance, and minimizing environmental leakage remains a crucial research objective.

Microcapsule technology offers a sophisticated encapsulation approach that effectively isolates core materials from the external environment within microsealed capsules, enabling controlled release under specific conditions to achieve preservation, safety, and performance enhancement objectives [20,21]. This versatile technology demonstrates extensive applicability across diverse industrial sectors, including pharmaceuticals, cosmetics, food additives, pesticides, industrial chemicals, and adhesive manufacturing [22]. Melamine-formaldehyde resin (MFR) has formed strong cross-linking bonds during the curing process, with only low formaldehyde release [23]. Although MFR lacks inherent anti-microbial properties, it exhibits exceptional thermal stability and demonstrates remarkable long-term storage stability, making it particularly suitable as an encapsulation material. These characteristics have led to its widespread application in the encapsulation of volatile compounds, including essential oils and agricultural pesticides [24,25]. Sui [26] effectively encapsulated water-soluble inorganic and organic active substances in melamine-formaldehyde-based microcapsules, studying the size distribution, encapsulation efficiency, payload, morphology, and release rate in aqueous environments. Similarly, Yan [27] employed MFR to microencapsulate pyraclostrobin, developing an environmentally compatible capsule suspension. F. Salaün [28] utilized n-hexadecane as the core material and melamine-formaldehyde as the shell, preparing microcapsules through in situ polymerization. Their research critically revealed that process parameters significantly influence microcapsules characteristics, including particle size, distribution, and surface morphology. Representative studies have demonstrated the practical applications of this technology. However, research on microencapsulating IPBC remains very limited, primarily due to the limited water solubility of IPBC. Selecting an appropriate emulsifier to better emulsify IPBC and prepare microcapsules with regular morphology and excellent sustained release performance, thereby extending the durability of IPBC, remains a significant challenge.

In this study, we successfully synthesized an IPBC-loaded microcapsule antifungal agent via in situ polymerization, utilizing MFR as the shell material. Microencapsulation not only provided a physical barrier for IPBC but also significantly enhanced its thermal stability and UV resistance, effectively extending the endurance of bamboo. This study comprehensively investigated the synthesis mechanism of microcapsules and evaluated their anti-mold performance against individual and mixed mold strains, simulating outdoor application. This innovative approach offers a promising solution to optimize IPBC applications while mitigating the potential adverse effects of biocides on human health and the environment. It also provides a novel long-term protective solution for biomass materials.

## 2. Materials and Methods

### 2.1. Materials

MFR was purchased from Hongming Chemical Reagent Co., Ltd., Jining, China. The antifungal agent 3-Iodo-2-PropynyI Butyl Carbamate (IPBC, C_8_H_12_INO_2_) was purchased from Aladdin Biochemical Technology Co., Ltd., Shanghai, China. Polysorbate 80 (Tween 80) and sodium dodecyl benzene sulfonate (SDBS) were purchased from Macleans Biochemical Technology Co., Ltd. in Shanghai, China. Sodium dodecyl sulfate (SDS) was purchased from Tianjin Guangfu Fine Chemical Research Institute, Tianjin, China. Sorbitan monooleate (Span 80, C_24_H_44_O_6_) was purchased from Tianjin Bodi Chemical Co., Ltd., Tianjin, China. Xylene (C_8_H_10_) and gum arabic power (GA) were obtained from Tianjin Zhiyuan Chemical Reagent Co., Ltd., Tianjin, China. Hydrochloric acid (HCl), and sodium hydroxide (NaOH) were supplied by Tianjin Kaitong Chemical Reagent Co., Ltd., Tianjin, China. All materials and chemicals were used as received without any further purification.

The 3-year-old moso bamboo (*Phyllostachys edulis*) was obtained from Huangshan, Anhui, China. We removed the bamboo inner skin and bamboo outer skin, took the middle part of bamboo culm wall and processed it into test samples with dimensions of 50 mm × 20 mm × 5 mm (L × W × T).

There were three molds, namely *Aspergillus niger V. Tiegh* (A. niger), *Trichoderma viride Pers. ex Fr.* (T. viride), and *Penicillam citrinum Thom* (P. citrinum), respectively, which were all provided by the Chinese Academy of Forestry.

### 2.2. Preparation of the Microcapsules

At room temperature, 0.25 g IPBC was completely dissolved in the tube containing 5 mL xylene solution. The solution was transferred to a 200 mL beaker, then added to 50 g deionized water before 1 wt% emulsifier was also added. The solution was sheared at 6000 r/min for 10 min using high-speed separation and a homogenizer to obtain milky white core material emulsion. We added 15 wt% MFR prepolymer solution to the emulsion, adjusted the pH to 3–4 using 1M hydrochloric acid solution, and heated to 50 °C using a constant-temperature magnetic stirrer at 400 r/min, continuing the reaction for 6 h. After the reaction, the solution was filtered and repeatedly washed with distilled water 3–5 times, with the resulting white filter cake serving as the microcapsules. The product was then dried in an oven at 50 °C for 48–72 h, ultimately yielding a white powder.

### 2.3. Mold Resistance Test

**Figure 1:**
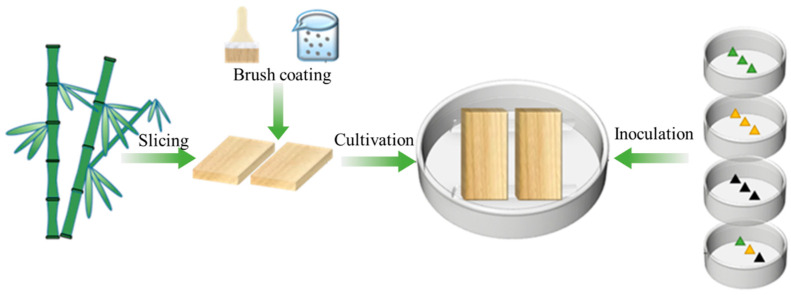
Schematic diagram of bamboo anti-mold testing treatment.

Mold resistance tests on treated bamboo samples were carried out according to the GB/T 18261-2013 “Test method for anti-mold agents in controlling wood mold and stain fungi” [29]. The bamboo anti-mold testing process is shown in Figure 1. The 30 wt% microcapsule solution was uniformly brushed onto the bamboo surface and air-dried naturally.

#### 2.3.1. Preparation of Fungal Mycelium and Spore Suspension

Under sterile conditions, fungal samples were taken using sterile needles and added to sterile water. They were then stirred in a homogenizer at 100 r/min for 10 min to produce the mycelium and spore suspension.

The three types of mold suspensions obtained by the method described above—A. niger, T. viride, and P. citrinum—were diluted using a McFarland (BASO) standard tube to (1–2) × 10^6^ cells/mL and then mixed in equal volumes to obtain a mixed mold suspension.

#### 2.3.2. Sample Inoculation and Cultivation

Under a laminar flow hood, the mycelium and spore suspension were taken using a pipette and injected into sterilized Petri dishes, which were then placed in an incubator with a constant temperature and humidity at conditions of 28 °C and 85% relative humidity for 7 days of sample inoculation. The bamboo samples were wrapped in multi-layer gauze and sterilized in an autoclave at 121 °C for 30 min. The microcapsules reagent was evenly brushed onto the surface of the bamboo samples, then air-dried and placed into Petri dishes already filled with mycelium, followed by incubation in a constant temperature and humidity incubator under the same conditions (28 °C and 85% relative humidity) for 4 weeks.

#### 2.3.3. Prevention and Treatment Effectiveness Calculation

The anti-mold levels were classified from 0 to 4 based on the extent of mold colonization (Table 1). The resistance efficiency (RE) against A. niger, T. viride, P. citrinum, and mixed mold species was calculated as follows:

(1) $$RE\;(\%)=\left(1-\frac{P_{1}}{P_{0}}\right)\times 100\%$$

where *P*_1_ is the percentage of fungal growth on the test block and *P*_0_ is the percentage of the fungal growth on the untreated controls [29].

### 2.4. Morphological Observations

We uniformly apply the sample to be tested on a glass slide and observe the microscopic scale formation rules and characteristics of microcapsules using an Optical Microscope (OM, BX53M, Olympus Corporation, Tokyo, Japan).

The microstructure of bamboo and microcapsules was observed using Scanning Electron Microscopy (SEM, Quanta-200, FEI Company, Hillsboro, OR, USA) and Transmission Electron Microscope (TEM, H-7650, JEOL, Tokyo, Japan). The completely dried microcapsules powder was mounted on the sample stage using conductive adhesive and subsequently coated with a thin gold layer. The acceleration voltage was set at 10–15 kV, and the working distance was maintained at 10 mm.

### 2.5. FTIR Spectroscopy

The chemical structure of the microcapsules was identified by Fourier Transform Infrared Spectroscopy (FTIR, Frontier, PerkinElmer, Waltham, MA, USA).

Each sample was prepared by mixing with potassium bromide and scanned 32 times in transmittance mode, with a resolution of 8 cm^−1^ over the wavenumber range of 4000–400 cm^−1^.

### 2.6. Thermogravimetric Analysis

The thermal stability of the microcapsules in a nitrogen atmosphere was measured by Thermogravimetric Analysis (TGA, TG209F1, NETZSCH, Hanau, Germany), with a heating rate of 10 °C/min from ambient temperature to 800 °C.

### 2.7. UV Performance Test

We used a UV weathering test chamber (UVA340, Kairuide, Jingmen, China) to conduct ultraviolet light exposure experiments on microcapsules, setting specific parameters of wavelength 340 nm, irradiance of 40 W/m^2^, and a total exposure time of 24 h.

### 2.8. Drug Loading and Encapsulation Rate Test

The drug loading is measured using an extraction method, with ethanol solution acting as the extractant. A specific amount of microcapsule samples are placed into a mortar, and the microcapsules are gradually crushed and rinsed with the extractant to ensure complete release of the core. The filtrate is collected and quantified using high-performance liquid chromatography based on Formula (2) to calculate the drug loading of the microcapsules.

(2) $$Drug\;loading\;(\%)=\frac{Mmd}{Mmm}\times 100\%$$

where *M_md_* is the measured drug mass and *M_mm_* is the mass of the microcapsules.

The encapsulation efficiency is calculated using the Formula (3):

(3) $$Encapsulation\;efficiency\;(\%)=\frac{Drug\;loading\;(\%)\times Mtmm}{Mtmd}\times 100\%$$

where *M_tmm_* is the total mass of the microcapsules and *M_tmd_* is the total mass of the drug used.

### 2.9. Sustained Release Performance Test

The microcapsules were placed in a dialysis bag (with a cutoff molecular weight of 3500 Da and a width of 24 mm), and ethanol was selected as the release medium. A magnetic stirrer operating at 150 r/min was employed to enhance the release process. At specified time intervals, a predetermined volume of the release medium was collected for quantification using high-performance liquid chromatography (HPLC) to evaluate the sustained release characteristics of the microcapsules.

The drug loading was quantified using high-performance liquid chromatography (Thermo TSQ Quantum Ultra, Thermo Fisher Scientific, Waltham, MA, USA). The analysis employed a Waters C18 column (100 mm × 2.1 mm, 1.7 μm) as the stationary phase, with the column temperature being maintained at 30 °C. The mobile phase comprised a 70:30 (*v*/*v*) mixture of methanol and water, with a flow rate of 1 mL/min and a detection wavelength of 200 nm. A sample of 10 mg was accurately weighed, dissolved in methanol, and diluted to a final volume of 10 mL. Subsequently, 1 mL of this solution was further diluted to 100 mL and filtered through a 0.22 µm membrane (diameter 25 mm, Bkman, Changsha, China). A calibration curve was constructed using standard solutions spanning a concentration range of 0.05–5 μg/mL, resulting in an R^2^ value of 0.999 or higher.

## 3. Result and Discussion

### 3.1. Morphological Analysis of Microcapsules with Different Emulsifiers

To explore the formation mechanism of microcapsules and prepare microcapsules with better morphology, we selected non-ionic surfactants Span 80, Tween 80, anionic surfactants SDS, SDBS, and natural polymer surfactant GA to investigate the impact of different types of emulsifiers on microcapsule preparation. Emulsifiers critically influence the successful preparation of microcapsules. Not only can they promote initial droplet formation during homogenization, but they also enhance droplet stability, facilitating prepolymer attraction and deposition on core particle surfaces [30,31]. Emulsifiers are surface-active amphiphilic molecules. Their molecular structure comprises both hydrophilic and lipophilic domains, enabling them to form oil–water interfaces during emulsification and prevent oil droplet aggregation [32]. The hydrophilic-lipophilic balance (HLB) value of a surfactant characterizes its affinity for oil or water [33]. Typically, a low HLB value indicates a lipophilic surfactant, while a high HLB value signifies a hydrophilic surfactant [34]. Most surfactants can be assigned a value representing their hydrophilic and lipophilic properties to determine the emulsion type. This simple index facilitates surfactant selection during emulsion preparation [35]. The HLB values of the five emulsifiers and their corresponding microcapsule preparation outcomes are comprehensively presented in Table 2.

**Figure 2:**
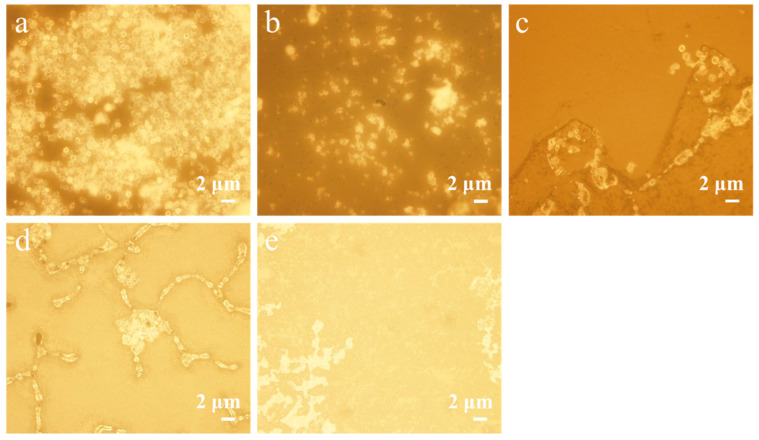
Optical microscope image of microcapsules: (**a**) Tween 80; (**b**) SDBS; (**c**) SDS; (**d**) GA; (**e**) Span 80.

As shown in Figure 2, we observed that, among the five tested emulsifiers, only Tween 80 and SDBS could successfully form microcapsules with superior morphological characteristics. The selective effectiveness of Tween 80 and SDBS suggests that their unique interfacial properties and molecular structures may play critical roles in achieving optimal microcapsule formation.

**Figure 3:**
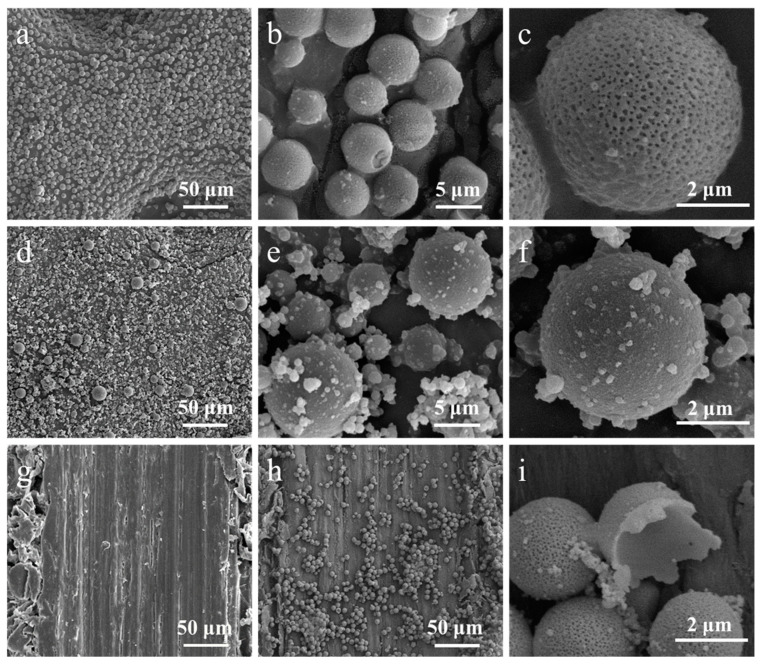
SEM images of microcapsules prepared with (**a**–**c**) Tween 80 and (**d**–**f**) SDBS; (**g**–**i**) SEM images of microcapsules prepared with Tween 80 coated on bamboo surface.

As shown in Figure 3, we examined the surface morphology of microcapsules prepared with emulsifiers Tween 80 and SDBS. The microcapsules produced with Tween 80 exhibited a uniform spherical shape, with a size of approximately 5 ± 1 μm, clear contours, and distinct porous characteristics on the surface (Figure 3a–c). Tween 80, as a non-ionic surfactant, forms stable emulsions at the oil–water interface, and its superior emulsification properties facilitate the evaporation of moisture during the curing process. Gas may accumulate within the capsules, potentially forming bubbles as water evaporates, which contributes to the development of a porous structure on the microcapsule surface. Additionally, Tween 80 may enhance the fluidity of the polymer chains, promoting the formation of more micro-sized pores. These structural features provide a strong foundation for the functionality and drug release properties of the microcapsules.

The microcapsules prepared with SDBS exhibited a regular spherical shape with a size range of 2–14 μm (Figure 3d–f). Although the surface shows no prominent porous features, numerous small resin particles were observed adhering to the microcapsule surface, resulting in increased surface roughness. The strong hydrophilicity and lipophilicity of SDBS create certain interactions between the aqueous and oily phases, inducing the formation of polymer particles and promoting their aggregation at the interface, thus facilitating the formation of microcapsules. These prominent nanoparticles can provide the additional interfacial area required for the microcapsules and may help improve adhesion to the bamboo substrate.

**Figure 4:**
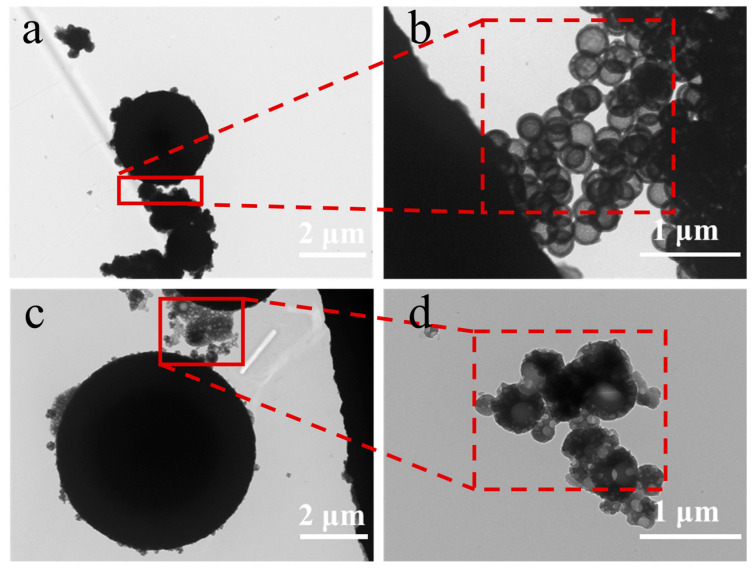
TEM images of microcapsules: (**a**,**b**) with emulsifier Tween 80; (**c**,**d**) with emulsifier SDBS.

TEM images illustrated the formation of primary particles on the surface of the microcapsules (Figure 4b,d), confirming their aggregation. The morphology of primary particles affects the surface morphology of microcapsules [36,37,38]. The microcapsules are formed through the precipitative polymerization of resin primary particles, which adhere to oil droplets and aggregate to create an interfacial network structure or porous layer around the template oil droplets. The process of microcapsules formation is dynamic and involves interfacial condensation that begins with monomer diffusion, followed by primary particle formation, phase separation, and shell growth, ultimately resulting in a dense microcapsule shell. The entire process is governed by interfacial reaction kinetics and self-limiting mechanisms, forming a continuous shell through the continuous stacking and merging of primary particles, with the reaction automatically terminating when the shell thickness reaches its limit [37]. Due to the inherent self-limiting nature of interfacial polymerization, void defects are formed in the microcapsules (Figure 3i).

Based on SEM and TEM results, we elucidated the mechanism of microcapsule formation. When IPBC was dissolved in xylene and mixed with the emulsifier solution, an oil-in-water (O/W) emulsion was formed. The hydrophilic polar groups of the emulsifier interact with water molecules, while the hydrophobic groups cover the surface of the core material droplets, providing a stable oil–water interface. The performance of emulsifier is independent of pH changes, allowing it to maintain emulsion stability under various conditions. When the pH of the system is adjusted to acidic levels, hydrogen ions can protonate certain groups within the MFR prepolymers, resulting in positively charged species. These positively charged polymers electrostatically adsorb onto the core droplets, gradually forming a layer of oligomeric film. As the condensation reaction continues, the molecular weight of the polymer increases, ultimately leading to the curing and cross-linking that forms a dense shell layer.

Overall, although the mechanisms by which these two emulsifiers form oil-in-water emulsions and microcapsules are fundamentally similar, their differing chemical properties and interactions result in distinct morphological characteristics of the final microcapsules. These differences will directly influence the performance of the microcapsules in drug delivery. In this study, considering the improvement of drug release efficiency and the protection of core materials, we selected microcapsules with a porous morphology, which better suits the research objectives and requirements.

### 3.2. FTIR Analysis of Microcapsules

**Figure 5:**
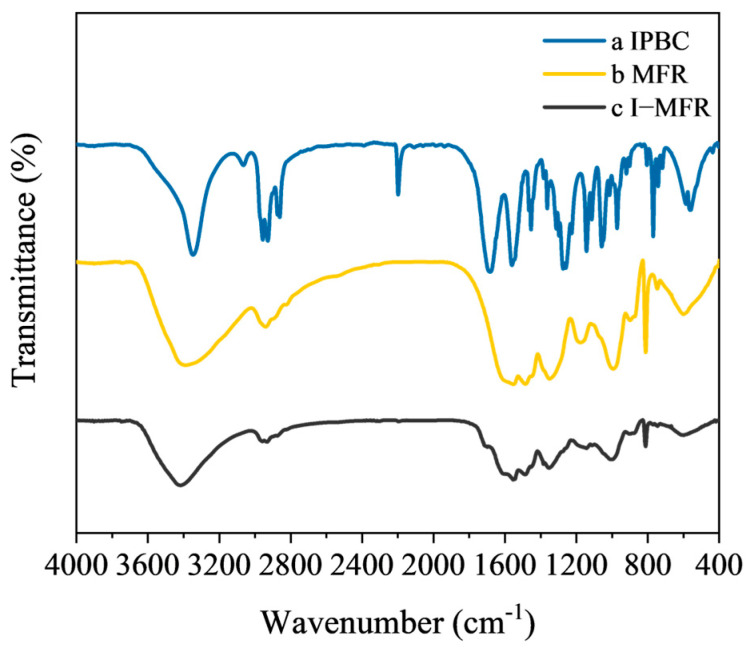
The FTIR spectra of (a) IPBC (core); (b) MFR (shell material); (c) I-MFR (microcapsules).

Figure 5 and Table 3 show the FTIR analysis results of the core material, shell material, and microcapsules. In MFR and I-MFR, the broad absorption peaks at approximately 3414 cm^−1^ and 3399 cm^−1^ were attributed to the superposition of N-H and O-H stretching vibrations [39,40]. Weak absorption bands at 2940 cm^−1^ and 2930 cm^−1^ corresponded to C-H stretching vibrations [41]. The peaks at 1557 cm^−1^ and 1553 cm^−1^ in MFR and I-MFR, along with the stretching vibration peak at 813 cm^−1^, represented the triazine ring C=N stretching and N-H shear bending vibrations, characteristic of MFR [39,42]. Absorption peaks at 1340 cm^−1^ and 1351 cm^−1^ indicated C-N stretching vibrations in tertiary amines [43]. The absorption peak at 2199 cm^−1^ in IPBC, originating from the alkynyl C≡C stretching vibration, and the similar peak at 2198 cm^−1^ in I-MFR, confirm the successful encapsulation of IPBC. FTIR analysis revealed that the characteristic peak distribution in I-MFR predominantly represents a superposition of peaks from IPBC and MFR, with no emergence of novel functional groups, indicating that IPBC was solely physically encapsulated within the melamine resin shell without inducing chemical reactions.

### 3.3. Thermal Stability Analysis of Microcapsules

**Figure 6:**
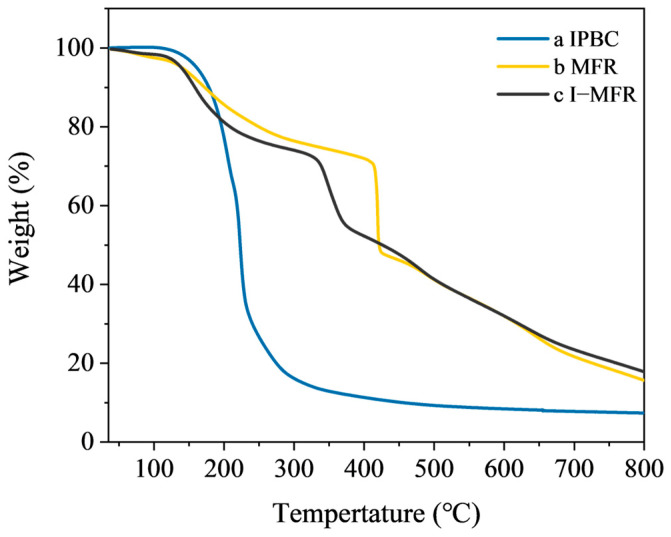
TG curves of (a) IPBC; (b) MFR; (c) I-MFR.

The TG curve of the microcapsules can be systematically divided into three distinct stages (Figure 6). Below 100 °C, a minor mass loss occurs due to the evaporation of adsorbed moisture [44]. Between 120 and 170 °C, volatile organic compounds, including free formaldehyde and trace amounts of the solvent xylene, are released from the MFR shell [45]. In the 300–400 °C range, significant degradation of the shell material is observed, characterized by the rupture of methylene bridges and ether bonds [46]. This degradation results in a mass loss of 20–30%, an accelerated decomposition rate, and a reduction in structural integrity. Subsequently, during the 400–800 °C phase, carbonization occurs, wherein residual organic materials continue to decompose, forming solid carbon residues. This stage results in an additional mass loss of approximately 30%, with the maximum degradation rate noted at around 350 °C. The principal decomposition of IPBC takes place between 150 and 300 °C, involving the thermal pyrolysis of halogenated hydrocarbon structures [19]. The thermal stability of the melamine-formaldehyde shell is crucial for protecting the core material in microcapsule applications. At 800 °C, the microcapsules retain approximately 17% of their original mass compared to pure IPBC, indicating that the dense cross-linked network of MFR offers excellent thermal stability, thereby enhancing the thermal stability of the encapsulated IPBC. The comparative TG curves confirm that IPBC was successfully encapsulated into MFR.

### 3.4. UV Resistance of Microcapsules

**Figure 7:**
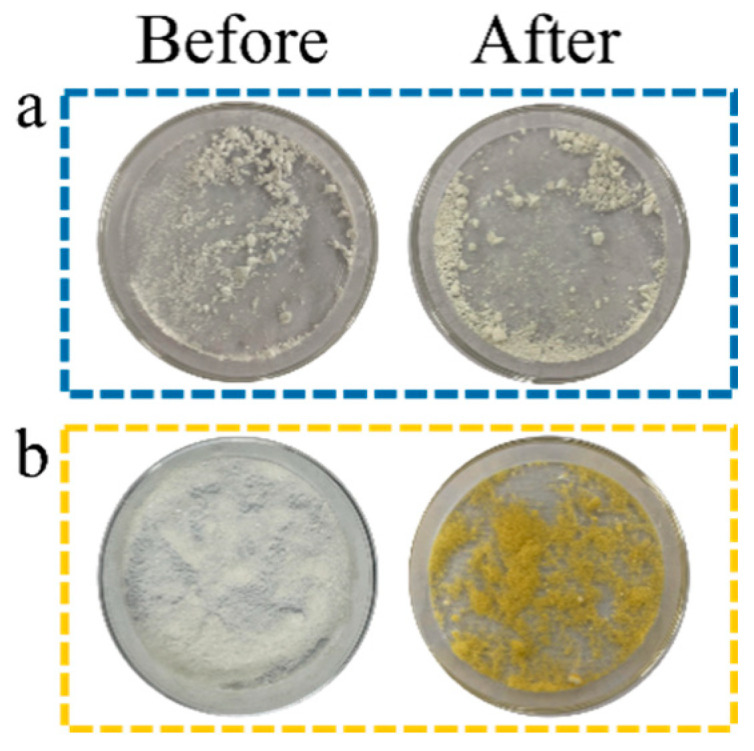
The results of UV irradiation treatment of (**a**) I-MFR and (**b**) IPBC.

Figure 7b demonstrates that IPBC exhibited a distinct color transition from white to yellow upon exposure to UV radiation, primarily due to photochemical oxidation reactions and molecular chain cleavage [47]. In comparison, I-MFR displayed minimal color alterations, suggesting that the MFR effectively mitigates UV-induced degradation of IPBC (Figure 7a). This protective mechanism significantly prolongs the anti-mold efficacy of IPBC, thereby contributing to the enhanced durability and long-term protection of bamboo.

### 3.5. Microcapsules Sustained Release Kinetics Analysis

**Figure 8:**
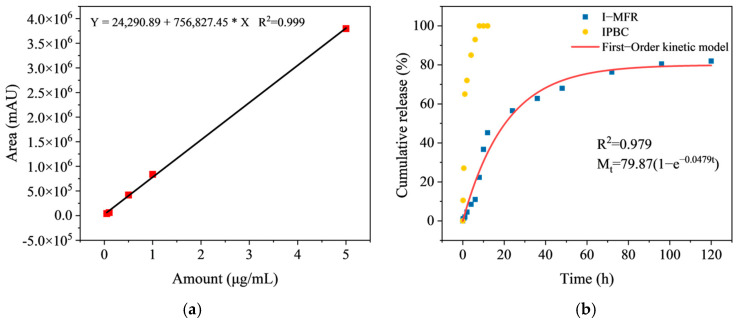
(**a**) Standard curve of IPBC; (**b**) release profiles of I-MFR and IPBC.

Figure 8b illustrates the temporal trend of cumulative release percentages for I-MFR and IPBC. The findings reveal that IPBC attained complete release within 20 h, whereas the cumulative release rate of I-MFR remained below 80% over a 120 h period. The First-Order Kinetic model was employed to assess the drug release kinetics of I-MFR, resulting in an R^2^ value of 0.979. This high value signifies that the fitted curve effectively characterizes the release dynamics of the drug from the microcapsules. The sustained release curve of I-MFR can be divided into three distinct stages. During the initial rapid release phase, the cumulative release within the first 8 h reached approximately 20–30% of the total drug release. This finding suggests that I-MFR displays a high initial release efficiency, enabling rapid inhibition of mold growth. In the stable mid-term phase, specifically between 12 and 48 h, the rate of increase in the released amount diminished, indicating that the microcapsules effectively attenuate the release rate of the fungicide, thereby prolonging its efficacy. Finally, in the subsequent phase (72–120 h), the cumulative drug release stabilizes, achieving 70–80% of the total release. This observation indicates that the drug release from the microcapsules has reached a state of equilibrium, thereby providing adequate release stability for extended anti-mold applications. HPLC was used to calculate the drug loading of the microcapsules at 20% and the encapsulation efficiency at 80%.

### 3.6. Analysis of Microcapsules Anti-Mold Performance

**Figure 9:**
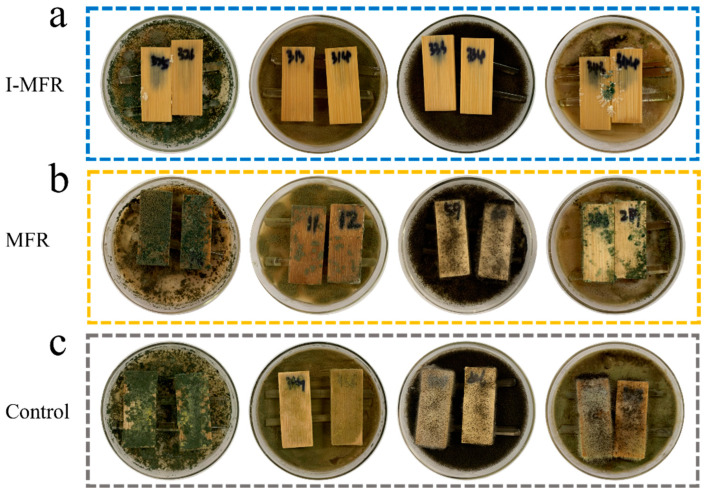
Mold infection results of bamboo samples after 4 weeks: (**a**) I-MFR; (**b**) MFR; (**c**) control group; mold species in order: T. viride, P. citrinum, A. niger, mixed mold.

Figure 9 illustrates the comparative anti-mold performance of the I-MFR, MFR, and control groups over 4 weeks. The results reveal that both the MFR group and the control group were entirely covered by T. viride, P. citrinum, A. niger, and mixed mold after 4 weeks, indicating that the MFR lacks anti-mold properties, similar to natural bamboo. In contrast, the I-MFR group exhibited excellent anti-mold performance, with only minor contamination by T. viride and mixed mold being observed on a few samples, while demonstrating complete inhibition of P. citrinum and A. niger. These findings align closely with the sustained release characteristics of I-MFR microcapsules observed in previous tests. The release mechanism operates in distinct phases: an initial rapid release effectively inhibits mold growth, a mid-term steady release maintains anti-mold efficacy, and a final slow release prolongs the protective duration, thereby achieving long-term anti-mold performance. This mechanism not only ensures a sustained effective concentration of the active components in the target area but also prevents drug waste and environmental pollution caused by single-dose releases. These findings provide a scientific basis for the long-term application of I-MFR microcapsules in bamboo protection and related fields, highlighting their broad prospects in anti-mold applications, particularly in industries such as furniture, construction, and coatings, where long-term mold protection is essential.

## 4. Conclusions

In this study, we investigated a preparation method for microencapsulated antifungal agents, specifically utilizing in situ polymerization to encapsulate the antifungal agent IPBC within MFR. We examined the effects of various emulsifiers on the morphology of the microcapsules and clarified the formation mechanism of these microcapsules. Our findings indicate that the emulsifier Tween 80 facilitates the formation of nanoscale micropores on the microcapsule’s surfaces. The porous morphology of the microcapsules is primarily established through the generation and deposition of emulsifier micelles. Compared to previously reported microcapsules, our porous microcapsules exhibit significant structural advantages: the nanoscale micropores exhibit a uniformly distributed pattern which not only enhances the release efficiency of the antifungal agent IPBC but also improves its thermal stability and UV resistance. This effectively boosts the anti-mold performance and durability of bamboo.

## Figures and Tables

**Table 1:** Anti-mold levels.

Value	Growth of Mycelium
0	No hypha growth on the surface of the specimen
1	Area of mycelial infection < 25%
2	25% < Area of mycelial infection < 50%
3	50% < Area of mycelial infection < 75%
4	Area of mycelial infection > 75%

**Table 2:** HLB Values of emulsifiers and experimental results.

Emulsifier	HLB	Results
Tween 80	15	The microcapsules have a porous surface, with a particle size of about 5 ± 1 μm, and the distribution is relatively uniform
SDBS	10.6	Resin particles are adhered to the surface of the microcapsules; the particle size is between 2 and 16 μm, and the size is relatively uneven
SDS	40	Uncapsulated
GA	——	Uncapsulated
Span 80	4.3	Uncapsulated

**Table 3:** An FTIR spectral characteristic table of functional groups.

Wavenumber (cm^−1^)	Functional Group	Vibration Type
3414/3399	N-H and O-H	Stretching vibrations
2940/2930	C-H	Stretching vibrations
2199/2198	Alkynyl C≡C	Stretching vibration
1557/1553	Triazine ring C=N	Stretching vibration and N-H shear bending vibrations
1340/1351	Tertiary amines C-N	Stretching vibrations
813	N-H	Shear bending vibrations

## Data Availability

The data are contained within the article.
